# Two-Tier Power and Energy Thresholds Governing Discharge Plasma-Induced Cell Death During Molecular and Gene Delivery

**DOI:** 10.3390/ijms27083606

**Published:** 2026-04-18

**Authors:** Taiki Hirohata, Hideki Motomura, Kazuki Tange, Yoshihisa Ikeda, Masafumi Jinno

**Affiliations:** Graduate School of Science and Engineering, Ehime University, Matsuyama 790-8577, Japan; n817001m@mails.cc.ehime-u.ac.jp (T.H.); motomura.hideki.mx@ehime-u.ac.jp (H.M.); tange.kazuki.nz@ehime-u.ac.jp (K.T.); ikeda.yoshihisa.dx@ehime-u.ac.jp (Y.I.)

**Keywords:** plasma mediated gene delivery, electrical equivalent circuit modeling, plasma-induced cell death mechanisms

## Abstract

This study investigates the mechanism of cell death associated with discharge plasma treatment from the perspective of electrical energy injection, using equivalent circuit network analysis to represent cells, buffer solutions, and well plates as electrical components. Our analysis demonstrated that the observed cell death cannot be adequately explained by a One-Step Model, which assumes that cell death occurs when the total injected electrical energy simply reaches a specific threshold. Accordingly, we proposed a Two-Step Model that explicitly incorporates biological tolerance to external stimuli. In this model, a stimulus accumulates only when the instantaneous power exceeds a primary threshold, and cell death is induced only when this accumulated stimulus surpasses a secondary threshold of energy. The proposed Two-Step Model successfully reproduced the experimental cell death data. These findings suggest that plasma-induced cell death is not a simple physical destruction process governed solely by cumulative energy, but instead reflects a biologically regulated response characterized by a specific power-dependent tolerance. Consequently, this Two-Step Model could provide a theoretical foundation for future optimization of delivery conditions for macromolecules such as messenger RNA (mRNA).

## 1. Introduction

The global success of messenger RNA (mRNA) vaccines against COVID-19 has clearly demonstrated the therapeutic potential of nucleic acid medicines. However, addressing the dual challenges of food insecurity driven by global population growth [1] and intractable diseases for which definitive treatments remain elusive requires more advanced approaches at the genetic level. To meet these demands, molecular delivery technologies into cells have become a central driver of progress across diverse fields, including basic biology, medicine, and agriculture [2,3,4,5,6,7,8,9,10,11]. To date, multiple delivery platforms, including viral vectors, non-viral vectors such as lipofection and lipid nanoparticles (LNPs), and physical methods such as electroporation, have been developed. Nevertheless, each method exhibits intrinsic limitations that hinder widespread practical application [2,3].

Viral vectors achieve high transduction efficiency and have therefore been extensively developed [12,13,14,15,16,17]; however, they are accompanied by significant safety concerns, particularly immunogenicity and insertional mutagenesis [3]. Chemical delivery approaches, such as lipofection, are generally considered safer, but their efficiency depends strongly on cell type, which creates substantial limitations for in vivo applications [2,18]. Electroporation is a physical delivery technique that induces transient nanopores in the cell membrane through the application of high-voltage pulses and can be applied to a wide range of molecular cargos [2,19]. However, because the operational window between reversible membrane permeabilization and irreversible membrane breakdown is extremely narrow, it remains difficult to simultaneously achieve high delivery efficiency and high cell viability [2,20].

In this context, following the discovery of discharge plasma-mediated molecular delivery [21,22,23], which originated from a technique utilizing cell membrane response under high-frequency high electric fields in the early 2000s [24], multiple research groups have reported various plasma-based delivery method [25,26,27,28,29,30,31,32]. Despite these developments, important challenges persist, including limited delivery efficiency, decreased cell viability caused by prolonged plasma exposure, and the absence of clear efficacy for large molecules such as plasmid DNA.

To overcome these limitations, we established a plasma molecular introduction method based on micro discharge plasma [33,34,35,36,37,38,39,40]. Plasma is capable of simultaneously providing multiple stimuli, including ions, electrons, radicals such as reactive oxygen and nitrogen species (RONS), and electric fields. We previously demonstrated that this combined stimulation activates endocytosis, thereby promoting molecular introduction into cells [36,37]. In addition, analysis using an equivalent electrical circuit network (EECN) model revealed that the dominant factor governing molecular introduction is the electrical current flowing into the cells [40]. This observation indicates that introduction efficiency can be improved through accurate control of electrical parameters. Although discharge plasma also generates chemical factors such as RONS, the present study focuses on quantifying the electrical power/energy injected into the cell layer using an EECN framework. Chemical effects are not modeled and are discussed as a limitation and avenue for future work in the Discussion. Accordingly, this study primarily aims to validate an EECN-based quantitative model of electrical dose, rather than to primarily focus on delivery protocol development/optimization. To rationally design plasma treatment conditions, it is essential to quantify the electrical “dose” delivered to the cell layer, defined as the spatial distribution of instantaneous power and accumulated energy that arise from the electrode configuration, medium properties, and cell-layer impedance. However, these quantities cannot be measured directly at the cell layer during plasma irradiation, and the observed outcomes, such as delivery efficiency and viability, represent only integrated biological responses. To bridge this gap, we employ an EECN model that discretizes the culture system into a network of impedances and enables calculation of the radial distributions of voltage, current, and the resulting power/energy injected into the cell layer. This EECN framework provides a quantitative link between controllable electrical parameters (applied voltage, frequency, and treatment duration) and local cellular stress, thereby enabling threshold-based modeling and experimental validation using the expansion of the cell-death radius.

One of the major remaining issues in plasma-based molecular introduction is cell death occurring near the plasma treatment site. Previous studies examining biomedical applications of atmospheric pressure plasma, including plasma jets, have reported that plasma exposure induces both apoptosis and necrosis, largely mediated by plasma-derived RONS. For example, Ahn et al. (2011) reported mitochondrial membrane depolarization and reactive oxygen species (ROS) induced apoptosis in HeLa cells treated with air and N_2_ plasma jets [41]. Similarly, Akter et al. (2020) demonstrated apoptosis induction in U87 MG brain tumor cells through ROS generation and activation of the p38/MAPK signaling pathway [42]. In addition, plasma-derived factors, including RONS, ultraviolet radiation, and charged particles, have been shown to damage not only cellular functions but also nucleic acids themselves, such as plasmid DNA, resulting in single strand and double strand breaks as well as base oxidation [43,44,45]. Other studies further suggest that the electric stimulation conditions used for molecular delivery, particularly pulsed electric fields, may contribute to nick formation or additional nucleic acid damage [46,47]. Therefore, for the practical implementation of plasma molecular introduction, treatment conditions must be carefully designed to minimize nucleic acid damage while preserving both high delivery efficiency and cell viability.

Our plasma treatment system employs a configuration in which a cell culture plate is placed on a grounded copper plate electrode, while a non-contact needle electrode is positioned above the plate. Discharge plasma is generated by applying a high voltage between the electrodes. A key feature of this system is that treatment is performed under conditions in which electrical current flows through the cells. Notably, Ninagawa et al. (2025) demonstrated that apoptosis induction in cancer cells by nanosecond pulsed electric fields (nsPEF) is quantitatively related to the amount of electrical stimulation, as revealed by frequency analysis [48]. Although understanding and controlling plasma-induced cell death is essential for biomedical applications, the underlying mechanisms remain insufficiently understood. In this study, we quantify the electrical dose associated with current flow during discharge treatment (instantaneous power and accumulated energy) and examine its relationship with the spatiotemporal progression of cell death.

The most intuitive hypothesis for cell death is thermal damage, exemplified by Joule heating, and is described by a One-Step Model in which cell death occurs once the accumulated energy exceeds a lethal threshold. However, biological systems naturally exhibit repair responses to a certain level of stress [49,50,51,52]. Given this biological reality, a more sophisticated modeling framework is required.

Building on the understanding that cellular responses often proceed in a stepwise manner [53,54,55,56], this study proposes and validates a Two-Step Model. In this framework, cell death is considered not as a single event but as a two-stage process. In the first stage, an initial response is triggered when the instantaneous power reaches a threshold that exceeds the cell’s immediate coping capacity. In the second stage, continued application of this intense stimulus above the threshold leads to the accumulation of electrical energy effective for cell death. Once this accumulation exceeds the cell’s repair limit, irreversible cell death occurs. In this study, we demonstrate the validity of the Two-Step Model by comparing experimental data describing the expansion of the cell death area radius as a function of plasma treatment time with calculated results obtained from both the One- and Two-Step models. This investigation provides a theoretical foundation for controlling cellular responses in next-generation plasma medicine.

## 2. Results

### 2.1. Experiment Validation of the Cell Death Area Evaluation Method

**Figure 1:**
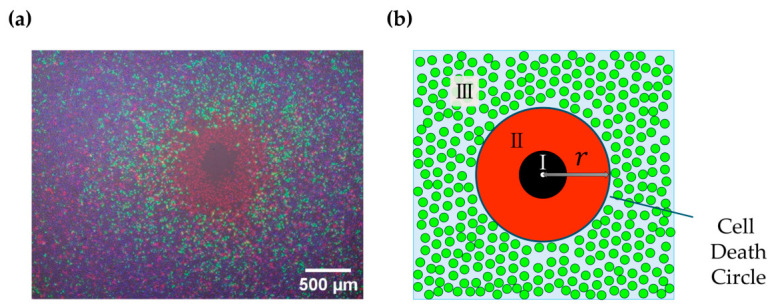
Visualization of cell death regions induced by plasma treatment. (**a**) A bright-field image and a merged fluorescence image (Hoechst 33342 for nuclei, PI for dead cells, and GFP for expression), illustrating three distinct regions. (**b**) A corresponding schematic representation. In Region III of the schematic, the light blue background represents the continuous layer of viable cells, and the scattered green circles indicate individual GFP-positive cells. Shown are: (I) Detachment zone, which appears black because cells have detached from the well surface, resulting in no detectable fluorescence. (II) Dead cell zone, in which cells are dead but remain adherent and therefore exhibit strong red fluorescence due to propidium iodide staining. (III) Viable transfection zone, where PI signals decrease, indicates fewer dead cells, while green fluorescence from GFP expression indicates successful plasmid delivery. The boundary between Region II and Region III defines the cell death circle.

In this study, we first verified the validity of the methodology used to evaluate the cell death area. Figure 1a shows a bright-field image and representative fluorescence microscopy images (Hoechst 33342/propidium iodide (PI)/green fluorescent protein (GFP)) obtained under typical experimental conditions. As shown in Figure 1b, cells in the plasma treated wells can be classified into three distinct regions, I, II, and III, according to the radial distance from the center, which corresponds to both the electrode position and the plasma generation site. Region I, the innermost region, appears black in the fluorescence images because cells have detached from the plate surface, resulting in the absence of detectable fluorescence signals. In Region II, the middle region, cells are dead but remain attached to the substrate and therefore exhibit red fluorescence due to propidium iodide, PI, staining. In Region III, the outermost region, PI red fluorescence decreases, indicating a rapid reduction in the number of dead cells, while green fluorescence is simultaneously observed, indicating successful introduction and expression of the GFP plasmid. Importantly, the radial position at which PI fluorescence begins to decrease coincides with the onset of GFP derived green fluorescence. Based on this correspondence, the radius of the circular cell death area was defined as the inner boundary of the GFP positive region, enabling stable and reproducible measurement of the cell death area. Within the field of view of the merged images (including bright-field, Hoechst 33342, PI, and GFP), GFP-negative but viable cells were not frequently observed on the Region II side. Accordingly, *r* represents the fluorescence-defined boundary between Regions II and III, rather than the detachment region (Region I). Therefore, in the following analyses, we treat the time-dependent change in *r* as the dynamics of the death-progression front (the II–III boundary), rather than as a description of cellular states across all inner regions.

### 2.2. Experiment (Treatment Time-Dependent Variation in the Radius of the Cell Death Circle)

**Figure 2:**
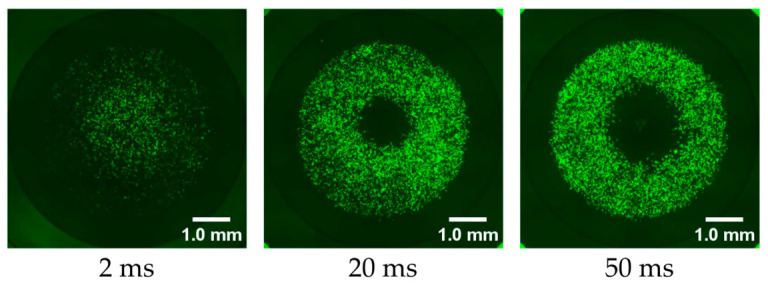
Representative fluorescence images obtained under typical plasma treatment conditions.

**Figure 3:**
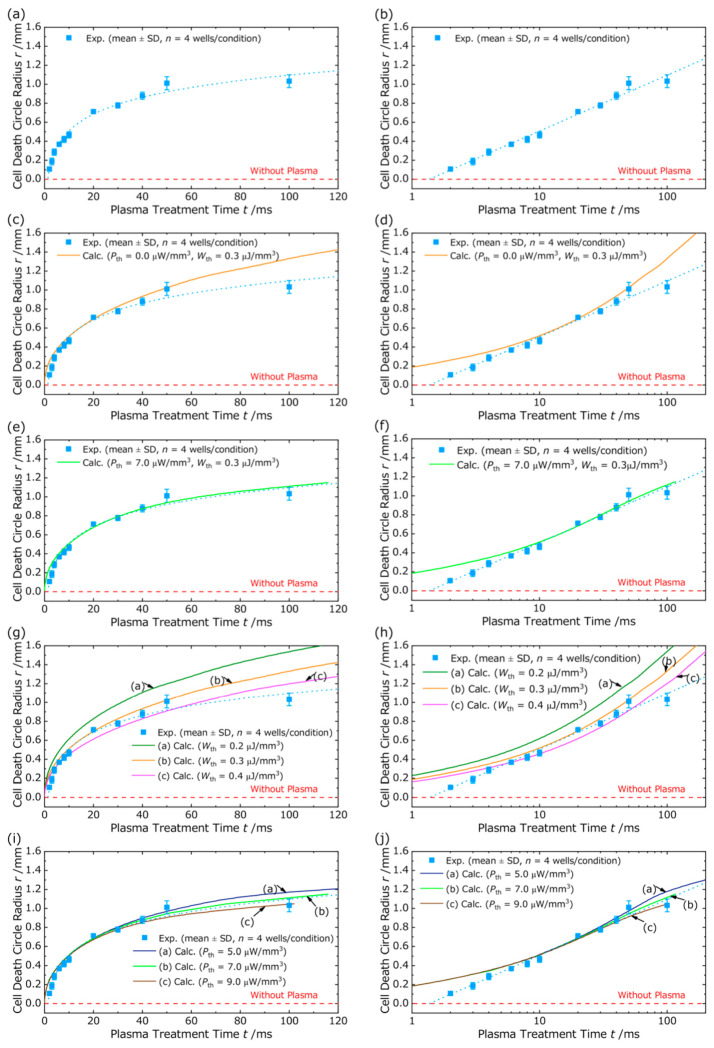
Experimental results, model reproduction, and parameter sensitivity of the cell-death radius as a function of plasma treatment duration. (**a**,**b**) Relationship between plasma treatment duration and the radius r of the cell-death area measured 24 h after treatment. Panels show r plotted as a function of treatment duration on (**a**) a linear scale and (**b**) a logarithmic scale. The radius r was quantified as the inner boundary of the GFP-positive region (i.e., the fluorescence-defined boundary between Regions II and III; see Figure 1 and Section 2.1). Each data point represents the mean of four measurements obtained from four wells per condition within a single experiment, with error bars indicating the standard deviation (SD, n = 4 wells per condition). (**c**,**d**) Comparison of experimental data and calculated results obtained using the One-Step Model for the cell-death radius *r* as a function of treatment duration, shown on (**c**) a linear and (**d**) a logarithmic horizontal axis. The thresholds are set as follows: $P_{th}$ = 0.0 $\mu W/{mm}^{3}$ and $W_{th}$ = 0.3 $\mu J/{mm}^{3}$. (**e**,**f**) Comparison of experimental data and calculated results obtained using the Two-Step Model for the cell-death radius *r* as a function of treatment duration, shown on (**e**) a linear and (**f**) a logarithmic horizontal axis. The thresholds are set to $P_{th}$ = 7.0 $\mu W/{mm}^{3}$ and $W_{th}$ = 0.3 $\mu J/{mm}^{3}$. (**g**,**h**) Sensitivity analysis: calculated curves obtained using the One-Step Model with varying $W_{th}$ thresholds, shown on (**g**) a linear and (**h**) a logarithmic horizontal axis. The values of $W_{th}$ are 0.2, 0.3, and 0.4 $\mu J/{mm}^{3}$. (**i**,**j**) Sensitivity analysis: calculated curves obtained using the Two-Step Model with varying $P_{th}$ thresholds at a fixed value of $W_{th}$, shown on (**i**) a linear and (**j**) a logarithmic horizontal axis. The values of $P_{th}$ are 5.0, 7.0, and 9.0 $\mu W/{mm}^{3}$.

Figure 2 shows GFP fluorescence images obtained under representative plasma treatment durations of 2, 20, and 50 ms. At the shortest treatment duration, 2 ms, GFP fluorescence was observed over nearly the entire well, and the radius of the cell death area was minimal. Figure 3 presents the relationship between the cell death area radius r and the plasma treatment duration measured 24 h after irradiation. Figure 3a and Figure 3b show this relationship using linear and logarithmic horizontal axes, respectively. As shown in Figure 3a, the radius r increased with increasing treatment duration in the range from 2 to 50 ms. However, when the treatment duration exceeded 50 ms, the expansion of r slowed, resulting in a more gradual increase. As illustrated in Figure 3b, when the data are plotted on a semi-logarithmic scale, the radius r shows a linear relationship with the logarithm of the plasma treatment time. Taken together, these results suggest that cell death is not simply determined by the cumulative time integral of plasma-derived stimuli, instead suggesting the presence of a stimulus intensity threshold required to induce cell death.

### 2.3. Calculation (Modeling of Cell Death Using Electric Equivalent Circuit Analysis)

#### 2.3.1. One-Step Model

Figure 3c,d shows the calculated treatment durations required to induce cell death at a radial distance r as a function of injected power, as determined by Equation (2), together with the corresponding experimental results. The horizontal axes in Figure 3c and Figure 3d are presented on linear and logarithmic scales, respectively. Although the model intrinsically calculates the time at which cells at a given radius reach cell death, the results are shown by relating the cell death radius to the treatment duration in order to allow direct comparison with the experimental observations. Under the assumption that cell death occurs when the total injected electrical energy reaches a predefined threshold $W_{th}$, the calculated curves deviated markedly from the experimental values for treatment durations longer than 20 ms, as shown in Figure 3c. In addition, the logarithmic plot in Figure 3d revealed clear discrepancies in both the short duration range, below 10 ms, and the long duration range, above 20 ms, indicating greater inconsistency than that observed in the linear representation. Notably, these discrepancies persisted in both linear and logarithmic plots despite adjusting $W_{th}$. In particular, within the framework of the one-step model, the calculated curves in the logarithmic representation consistently exhibited a downward convex shape over the experimental time range, regardless of the parameter settings. This is discussed further in Figure 3g,h.

#### 2.3.2. Two-Step Model

Based on Equation (5), we calculated the time required to induce cell death at a radial distance r through the injection of electrical energy effective for cell death, and the results were plotted together with the experimental data in Figure 3e,f. Figure 3e and Figure 3f present these results using linear and logarithmic horizontal axes, respectively. Under the hypothesis that cell death is initiated when the instantaneous power injected into a cell exceeds a threshold $P_{th}$ for a sustained period and that the accumulated stimulus subsequently exceeds a secondary threshold $W_{th}$, calculations were performed by varying these two threshold values. As shown in Figure 3e,f, when $P_{th}=7.0\;\mu W/{mm}^{3},\;W_{th}=0.3\;\mu J/{mm}^{3}$, the calculated curves showed good agreement with the experimental data for treatment durations longer than 10 ms on both linear and logarithmic scales. In contrast, deviations between the model predictions and experimental results were observed for treatment durations of 10 ms or less. The sensitivity of the calculated curves to changes in $P_{th}$ and $W_{th}$ is further described in Figure 3i,j.

## 3. Discussion

**Figure 4:**
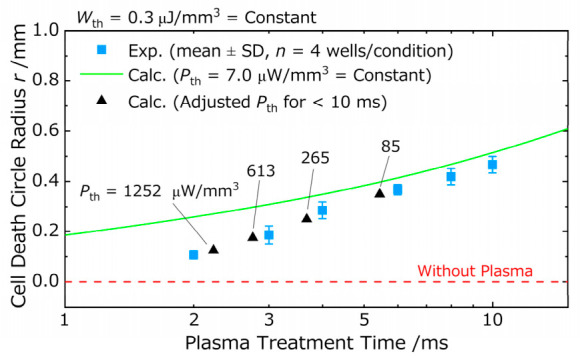
Supplementary analysis of the cell-death boundary considering the stimulus-duration dependency of the instantaneous power threshold. The One-Step Model with a constant threshold (green line) tends to overestimate the cell-death radius in the short-duration regime. This discrepancy suggests that cells may possess a specific tolerance toward abrupt, high-intensity stimuli. The black triangles (▲) represent recalculated points where the instantaneous power threshold ($P_{th}$) was individually adjusted to replicate the experimental cell-death boundary. The requirement to set a higher effective $P_{th}$ for shorter durations, particularly below the boundary of r ≈0.45 mm, indicates that cellular response characteristics vary depending on the duration of the stimulus. These results support the hypothesis that the evaluation of cell death under short-duration irradiation should consider not only cumulative energy but also the threshold characteristics of instantaneous power that may be dependent on stimulus duration.

In this study, we conducted a numerical analysis based on an EECN model to investigate the expansion of the cell death area induced by discharge plasma treatment and to compare the calculated results with experimental observations. The One-Step Model, which assumes that cell death occurs once a total electrical energy threshold is reached, failed to reproduce the experimental results in the long treatment duration range. Furthermore, when examined on a logarithmic scale, the calculated curves generated by the One-Step Model failed to capture the overall trend of the experimental data and differed from the approximately linear trend observed experimentally, regardless of parameter values. These results suggest that a model relying solely on cumulative energy is insufficient to explain the progression of plasma-induced cell death. In contrast, the Two-Step Model, which incorporates an instantaneous power threshold $P_{th}\;$ and a cumulative stimulus threshold $W_{th}$, showed good agreement with the experimental data, suggesting that plasma-induced cell death is governed by a two-stage biological response process. Nevertheless, discrepancies between the calculated curves and experimental values were observed for short treatment durations of 10 ms or less. This observation suggests that cells may exhibit resistance or latency in sensing short-term electrical stimuli. Specifically, the instantaneous power threshold $P_{th}$ required to induce cell death may increase as the stimulus duration decreases. Therefore, it may be necessary to treat $P_{th}$ as a time-dependent function, $P_{th}(t)$, that increases as the stimulus duration decreases. However, because the current Two-Step Model treats $P_{th}$ as a constant parameter, it cannot capture this temporal variation in resistance. As a result, the model likely underestimates the effective threshold immediately after voltage application, leading to an overestimation of the cell death area at very short treatment durations. To further examine this possibility, we conducted a supplementary analysis focusing on the short-duration regime (≤10 ms). In this analysis, we treated the instantaneous power threshold as potentially dependent on stimulus duration, and empirically determined $P_{th}\;$ such that it increased as the treatment duration became shorter. Using the experimentally observed radius r  within the ≤10 ms range, we estimated the effective $P_{th}\;$ required for cells at each radius to reach the lethal condition, and then recalculated the corresponding time-to-death. By introducing this stimulus-duration-dependent threshold, the agreement between the model and the experimental data in the ≤10 ms regime was markedly improved. The detailed procedure and the corresponding plots are provided in Figure 4.

The underlying concept of this model, which evaluates both stimulus intensity and cumulative dose, is consistent with principles observed in various biological systems. For example, during electroporation (EP), reversible nanopores form when the membrane potential exceeds a specific threshold, representing the first stage, and sustained stimulation then leads to the accumulation of membrane damage, ultimately resulting in irreversible cell death as the second stage [53,54]. Previous studies have also shown that threshold curves for electric field induced cytotoxicity display inflection points that depend on pulse conditions, indicating that a single mechanism cannot account for the full range of cellular responses [54]. Similar principles are observed in mechanical stress responses, where vascular endothelial cells adapt to moderate shear stress generated by blood flow but undergo apoptosis when excessive stress persists, thereby contributing to atherosclerosis [57]. In other stress modalities, including radiotherapy and chemotherapy, cells initially activate DNA repair and defense mechanisms as a first stage response. However, when accumulated damage exceeds the repair capacity, cells undergo active cell death through apoptosis as a second stage response [55,56,58,59,60]. Although these analogies do not directly validate the mechanism proposed here, they support the plausibility of distinguishing between a stimulus-intensity condition for initiating death-related responses and a cumulative condition for commitment to irreversible cell death.

From this perspective, the two thresholds in the present model may be interpreted in a general biological sense as representing two different requirements for cell death progression. The first threshold, $P_{th}$, corresponds to the level of instantaneous power that exceeds the capacity of cellular homeostatic mechanisms, including self repair and stress response systems. This threshold can be interpreted as a stimulus intensity threshold, at which cells transition from a state of repairable stress to a biological switching point where irreversible damage begins to accumulate. The second threshold, $W_{th}$, represents the cumulative dose at which this irreversible damage reaches a critical level and ultimately activates programmed cell death pathways such as apoptosis. It should be noted that in Region I, as defined in the Results, where cells detach near the center of the well, the precise cellular state remains unclear. Therefore, it cannot be conclusively determined whether cell death in this region results from a regulated two-stage response or from immediate physical destruction, such as necrosis, characteristic of a one stage process.

To examine whether the estimated $W_{th}$ value (0.3 μJ/mm^3^) is physically reasonable, we compared it with previously reported energy-density scales associated with other cell-death-inducing modalities. Regarding thermal damage, Morino et al. (2020) reported that an energy density of 2100–2500 J/cm^3^ (sum of three treatments) was required for complete tumor regression in magnetic induction hyperthermia [61]. In addition, in the context of irreversible electroporation (IRE), the apoptosis-inducing threshold in rat liver tissue has been reported as $5.9\times 10^{5}\;$J/m^3^ ($5.9\times 10^{-4}$ J/mm^3^) [62]. The estimated $W_{th}\;$ in the present study is therefore substantially lower than these reported values. These comparisons do not provide direct validation of the biological interpretation of $W_{th}\;$; rather, they serve as order-of-magnitude contextualization showing that the fitted value lies in a physically non-catastrophic range, far below energy scales typically associated with primary thermal damage or massive membrane breakdown. In this sense, the estimated threshold is not obviously physically implausible, although its direct biological correspondence remains unverified. Therefore, the absolute numerical value of $W_{th}\;$ should not be overinterpreted at this stage; rather, its significance lies primarily in supporting the qualitative need for a cumulative threshold in addition to an instantaneous one. Although the estimated energy density of 0.3 μJ/mm^3^ is well below the threshold reported for irreversible electroporation (IRE), this does not necessarily exclude downstream intracellular responses. Previous nsPEF studies reported intracellular ${Ca}^{2+}$ responses below the threshold for plasma membrane electroporation and in the absence of classical plasma membrane electroporation [63]. Therefore, the calculated micro-energy threshold ($W_{th}$) may be interpreted not as an indicator of physical membrane destruction itself, but as a phenomenological threshold associated with downstream intracellular responses.

While the precise mechanism by which physical stimulation is transduced into biochemical signaling has not been directly established in this study, previous findings allow us to propose a biologically plausible working hypothesis. Previous studies have shown that intracellular ROS and Ca^2+^ signals can mutually amplify one another through crosstalk, promoting Ca^2+^ release from the endoplasmic reticulum (ER) and mitochondrial ROS production, which can ultimately lead to apoptotic cell death [64]. In addition, the mitochondrial permeability transition pore (mPTP) is highly sensitive to Ca^2+^ overload and oxidative stress, and its sustained opening is known to trigger apoptotic or necrotic cell death [65]. In our previous studies, we reported that plasma treatment induces an increase in intracellular Ca^2+^ concentration even in an extracellular Ca^2+^-free environment [38], and that plasma stimulation is highly likely to generate intracellular ROS [66]. Taken together, these findings suggest that, in our current system, the electrical current flowing into cells during plasma stimulation may trigger both intracellular Ca^2+^ elevation and ROS production.

Based on these findings, $P_{th}$ may be interpreted as the threshold electrical stimulus intensity required to overcome the cellular buffering capacity and initiate death-related intracellular responses, such as endogenous ROS generation and intracellular Ca^2+^ signaling. Likewise, because $W_{th}$ is defined as the time integral of the instantaneous power exceeding $P_{th}$, it may be interpreted as the accumulation of effective stimuli required to commit the cell to irreversible death. From a biological perspective, this accumulated stimulus may correspond to integrated consequences of sustained intracellular stress, potentially including prolonged mPTP opening. However, it is important to emphasize that ${\;P}_{th}$ and $W_{th}$ are effective macroscopic parameters derived from the present EECN-based phenomenological model, rather than direct measurements of the intracellular energetic requirements for ROS production, Ca^2+^ flux, or mPTP opening. Their fitted values may also depend on how accurately the actual instantaneous plasma power delivered to cells is represented in the model; since the actual injected power may involve sharper transient peaks than assumed in this study, the estimated values of $P_{th}$ and $W_{th}$ could change if such characteristics are incorporated. Accordingly, the proposed ROS/Ca^2+^/mPTP interpretation should be regarded as an indirect inference and a working hypothesis, not as a quantitatively validated one-to-one mechanistic explanation.

These interpretations provide a logical link between the two macroscopic electrical thresholds derived from our model and the underlying microscopic biological responses, and they are broadly consistent with existing biological knowledge. However, we emphasize that this connection remains an indirect inference and a working hypothesis. Further experimental validation will be necessary to directly clarify the causal relationship between the proposed electrical stimulation parameters and the intracellular signal transduction pathways leading to cell death.

Although this study focuses on the electrical components of plasma stimulation, cell death is likely influenced by multiple factors, including reactive oxygen species (ROS) and intracellular ion dynamics [67,68]. In our microdischarge plasma-based delivery system, Ikeda et al. reported that H_2_O_2_ is one of the main contributors to gene transfection after plasma irradiation; moreover, exogenous addition of H_2_O_2_ up to 1 ppm did not markedly change the transfection efficiency or viability, suggesting that the amount of H_2_O_2_ generated by plasma irradiation is sufficient for transfection [34]. In contrast, excessive additional H_2_O_2_ (e.g., >1 ppm) increased cell death and reduced transfection efficiency [34]. Together with our previous EECN-based analysis indicating that molecular introduction in this system is dominantly governed by the electrical current flowing into cells [40], these observations suggest that, under the present treatment conditions (2–100 ms), ROS are unlikely to be the dominant factor determining the applicability of the EECN-based Two-Step model. However, for longer exposures or conditions leading to higher ROS accumulation, chemical effects may become non-negligible, and an integrated electrochemical model that couples electrical and chemical actions will likely be required. In addition, reactive nitrogen species (RNS) were not quantitatively evaluated in this study, and clarifying their contribution remains an important subject for future work.

In the present EECN analysis, the plasma path was approximated as a perfect conductor to reduce computational cost and to focus on the spatial distribution of electrical power/energy delivered to the cell layer. However, atmospheric-pressure discharges can exhibit nonlinear and time-varying electrical behavior, and more elaborate plasma equivalent-circuit models have been proposed [69]. Moreover, the actual discharge current may deviate from an ideal sinusoid and include sharp, spike-like transient components. Because these transients possess extremely high frequencies, they reduce the capacitive impedance of the cell layer.

Consequently, the actual local current and injected power may be underestimated in the current sinusoidal approximation model. If a more refined plasma model that faithfully reproduces these waveforms is constructed, it is expected to show that the instantaneous current flowing into the cells increases due to these spikes. Resolving such fast components requires high-bandwidth diagnostics, which remains an important topic for future work.

Therefore, the estimated thresholds (e.g., $P_{th}$ and $W_{th}$) should currently be interpreted as effective parameters under the present approximation. It is expected that integrating waveform-resolved plasma modeling in the future will enable a more quantitative discussion regarding plasma-induced cell death.

In the present Two-Step Model, the cumulative stimulus w(r) increases only during the time intervals when the instantaneous power p(r,t) exceeds the threshold $P_{th}$, and cell death is predicted when $w(r)\geq W_{th}$. This definition implies that, when considering treatment durations longer than those examined in this study (2–100 ms), including >0.1 s, the predicted increase in the cell-death radius should progressively slow because peripheral regions experience fewer/shorter $p(r,t)>P_{th}$ intervals and thus require longer times to reach $W_{th}$. Moreover, if p(r,t) remains below $P_{th}$ throughout the entire waveform at a given radius, w(r) does not accumulate there, suggesting that the predicted radius may approach an upper limit within this model framework.

In contrast, plasma treatments on the seconds-to-minutes scale commonly reported in the literature may involve non-electrical contributions such as the generation/accumulation of reactive species and bulk heating of the medium or tissue. Accordingly, the present model is positioned as a framework primarily intended to quantify cell-death processes driven by electrical stimulation under short-duration, high-current-density conditions, and addressing longer time scales will likely require integrating electrical, chemical, and thermal effects. The model is based on an in vitro single cell population and may not fully represent responses in different cell types or in three dimensional tissue environments. In addition, the values of $P_{th}$ and $W_{th}$ depend on the specific experimental conditions, and their physiological relevance requires further validation. The integration of time resolved measurements of membrane potential and intracellular ion dynamics with mathematical modeling will be essential for elucidating the biological basis of the Two-Step Model. Future studies using molecular markers to identify specific cell death modes, such as apoptosis and necrosis, are required to further clarify this biological basis. Clarifying the generality of these thresholds and their variation among cell types will contribute to the optimization of plasma treatment conditions and improved control of cell selectivity, which may lead to safer and more efficient applications in plasma-based molecular delivery and cancer therapy.

## 4. Materials and Methods

### 4.1. Experimental Setup

**Figure 5:**
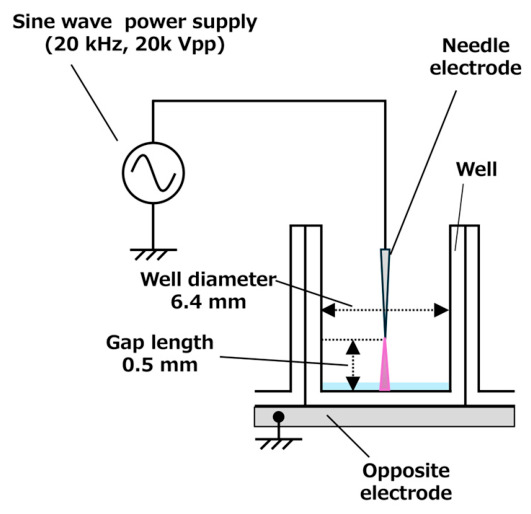
Schematic of the plasma treatment setup. A sinusoidal high-voltage power supply operating at 20 kHz and 20 kVpp is applied to a needle electrode to generate microplasma (purple) above the solution surface. The light blue region represents the solution, which is added dropwise onto cells in a 96-well plate. The bottom of the well is placed on a grounded electrode, ensuring that treatment occurs under conditions in which electrical current flows through the electrode, solution, and cells. The electrode gap length is 0.5 mm, and the well diameter is 6.4 mm.

The detailed configuration of the plasma molecular introduction system has been described previously [36]; therefore, only a brief overview is provided here. Figure 5 shows the configuration of the plasma treatment unit. A 96-well plate (IWAKI, Shizuoka, Japan) was placed on a grounded copper plate electrode. A micro high-voltage needle electrode was positioned at the center above each well, with a gap distance of 0.5 mm.

Cells were cultured on the bottom surface of each well and prepared at a confluent state, approximately 100% confluence. A typical bright-field image of the cell layer before plasma treatment is provided in Appendix A to document the approximately confluent condition. The cell line used in this study was NCTC clone 929, a mouse fibroblast subclone (JCRB Cell Bank, Osaka, Japan; Catalog No. IFO50409; Lot No. 10062023). According to the supplier information, these cells exhibit fibroblast-like morphology and tested negative for bacterial, fungal, and mycoplasma contamination. Cells were maintained in Eagle’s Minimum Essential Medium supplemented with 10% fetal bovine serum (FBS; Biowest, Cat. #S1400 - 500, Lot No. S00MG, Nuaillé, France,) at 37 °C in a 5% CO_2_ atmosphere. Subculturing was performed using 0.25% trypsin and 0.02% EDTA for cell dissociation, and no antibiotics were used. All cultures were maintained in a humidified incubator at 37 °C with 5% CO_2_. The plasmid DNA used in this study was pAcGFP1-N1 (4726 bp; Takara Bio, Shiga, Japan), which encodes the *AcGFP* gene

Before plasma irradiation, the culture medium was removed, and 4 μg of plasmid DNA dispersed in 4 μL of a PBS water buffer solution was added dropwise onto the cells. To generate microdischarge plasma at the tip of the high-voltage electrode, a sinusoidal voltage with an amplitude of 20 kV peak to peak and a frequency of 20 kHz was applied. The microplasma was irradiated onto the PBS solution for treatment durations of 2, 3, 4, 6, 8, 10, 20, 30, 40, 50, and 100 ms, after which 100 μL of culture medium was added. Following treatment, the samples were incubated for 24 h and subsequently observed by fluorescence microscopy.

To evaluate gene delivery, both bright-field and fluorescence images (GFP, PI, and Hoechst 33342) were acquired using a fluorescence microscope (BZ-X810, Keyence, Osaka, Japan). The radius of the cell death area was calculated using the integrated analysis software provided with the BZ-X800 system (version 1.1.1.8).

### 4.2. Electrical Equivalent Circuit Model

**Figure 6:**
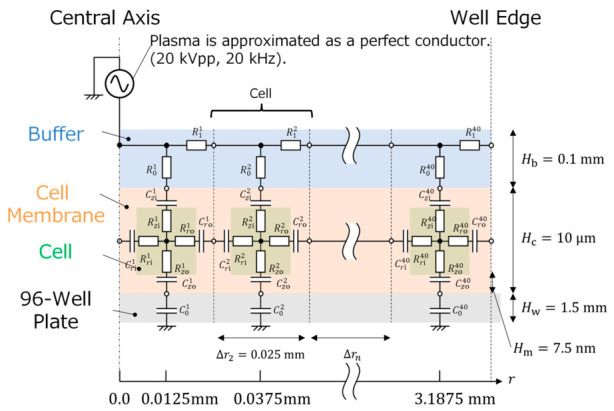
Experimental equivalent circuit model.

Details concerning the determination of circuit element constants and the analytical methodology of the EECN are described in reference [40]. The EECN analysis was performed using LTspice XVII (x64), (version 17.0.36.0; Analog Devices, Inc., Wilmington, MA, USA). As shown in Figure 6, the experimental system, consisting of the cells, the buffer solution, and the bottom plate of the well, was represented using an equivalent circuit model. In this study, axial symmetry was assumed for the spatial distributions of voltage and electrical current within the well. The computational domain was defined along the radial direction, extending from the center of the well to the region adjacent to the well wall. This domain was divided radially into 40 concentric regions, numbered from *n* = 1 at the center to *n* = 40 at the outermost periphery. To accurately capture the physical phenomena within the well, the radial segmentation width $\Delta r_{n}$ was non-uniformly distributed. A finer mesh was implemented near the center of the well (*n* = 1 to 4) to provide sufficient spatial resolution for the steep electric field gradients occurring near the needle electrode axis. Similarly, the resolutions near the well wall (*n* = 37 to 40) were more densely segmented to account for boundary effects.

Thus, given the well radius R, the segmentation width $\Delta r_{n}\;$ of each region was defined as follows:

- $For\;n=1,2,3,4\;and\;n=37,38,39,40:\Delta r_{n}=R/128$;
- $For\;n=5,6\;and\;n=35,36:\Delta r_{n}=R/64$;
- $For\;n=7\;to\;n=34:\Delta r_{n}=R/32$.

Overall, this spatial discretization enables detailed analysis both near the singularity at the center of the well and in peripheral regions where boundary conditions are applied.

The plasma was approximated as a perfect conductor. This simplified approximation was adopted to reduce computational cost and to focus on the spatial distribution of electrically injected power/energy into cells. The power supply was connected in series with the first unit cell of the buffer layer. The buffer layer consisted of a mixture of PBS, water, and plasmid DNA and had an electrical conductivity of 0.172 S/m. Accordingly, the conductivity of the buffer layer in the model was set to 0.172 S/m. Parameters for the remaining circuit elements are summarized in Table 1 [70,71,72], where *ε* denotes the vacuum permittivity. Using these parameters, the electrical current in each unit cell was calculated, and the intracellular current density was obtained by dividing the current by the corresponding cross sectional area.

### 4.3. Threshold-Based Cell Death Model

#### 4.3.1. Mathematical Definition of the One-Step Model

The instantaneous power p injected per unit volume into a cell located at a radial distance r from the center of the well, as well as the total energy w injected during the plasma treatment time, were calculated. Based on the EECN analysis, the current density J flowing through the cytoplasm was defined as the magnitude of the vector obtained by combining the radial and vertical current components. As shown in Equation (1), the instantaneous power per unit volume $p\left(r,t\right)$ was calculated using the electrical conductivity σ and the current density $J\left(r,t\right)$. The total injected energy $w\left(r\right)$ was then determined by integrating the instantaneous power over the plasma treatment duration $T_{p}$.

(1) $$w\left(r\right)=\int_{0}^{T_{p}}p\left(r,t\right)dt=\int_{0}^{T_{p}}\frac{J{\left(r,t\right)}^{2}}{\sigma }dt$$

where σ is the electrical conductivity of the cytoplasm. Since p(r,t) fluctuates within each cycle, we used the instantaneous waveform to define the exceedance duration.

Next, we determined the time required for $w\left(r\right)$ to reach a predefined threshold $W_{th}$ as shown in Equation (2). This duration was defined as $T_{One-Step}^{*}\left(r\right)$. It represents the time until cell death is induced by the injected energy at a distance r from the center.

(2) $$T_{One-Step}^{*}\left(r\right)=inf\left\{t\in \left[0,T_{p}\right]\;\left|\int_{0}^{t}p\left(r,t\right)dt\geq W_{th}\right.\right\}$$

#### 4.3.2. Mathematical Definition of the Two-Step Model

In the Two-Step Model, the calculation of the instantaneous power p per unit volume is identical to that in the One-Step Model (Section 2.3.1). Specifically, we determined p based on the electric field and current density applied to the cytoplasm at a distance r. However, the integration was performed only for the periods during which the instantaneous power p exceeded a predefined threshold $p_{th}$, as shown in Equation (3).

(3) $$w\left(r\right)=\int_{0}^{T_{p}}p\left(r,t\right)H\left(p\left(r,t\right)-P_{th}\right)dt$$

Here, $H\left(x\right)$ represents the Heaviside step function, defined in this study as follows:

(4) $$H\left(x\right)=1\left(x\geq 0\right),0\left(x<0\right)$$

Finally, we calculated the time at which this integrated value reached another threshold $W_{th}$. This duration was defined as $T_{Two-Step}^{*}$, the time until cell death is determined by the effective electrical energy, and is shown in Equation (5). All thresholds were determined by fitting the model to the experimental data. Across all treatment durations, we varied $P_{th}$ and $W_{th}$ and selected the parameter set that best reproduced the overall experimental trend on both linear and logarithmic time axes.

(5) $$T_{Two-Step}^{*}\left(r\right)=\inf\left\{t\in \left[0,T_{p}\right]|\int_{0}^{t}p\left(r,t^{\prime }\right)H\left(p\left(r,t^{\prime }\right)-P_{th}\right)dt^{\prime }\geq W_{th}\right\}$$

## 5. Conclusions

In this study, we examined the dependence of the circular cell death area radius on treatment time by calculating the electrical power and energy delivered to cells using EECN analysis and by directly comparing these calculations with experimental observations. The One-Step Model, which is based solely on cumulative energy dosage, failed to reproduce the experimental data for longer treatment durations. Moreover, when evaluated on a logarithmic scale, the calculated curves generated by the One-Step Model consistently exhibited a downward convex shape within the experimental time range, markedly differing from the linear trend observed experimentally.

By contrast, the Two-Step Model, which incorporates both an instantaneous power threshold ($P_{th}$) and a cumulative stimulus effective for cell death ($W_{th}$), successfully reproduced the experimental results across a wide range of treatment durations. This outcome demonstrates that plasma-induced cell death is governed by a two-stage, power dependent response rather than by a simple accumulation of total energy. This reflects universal biological responses to external stimuli, characterized by distinct thresholds for stimulus intensity and cumulative dose.

Although we initially hypothesized that cell death during plasma mediated gene delivery would result from physical necrosis caused by structural damage, the EECN-based analysis instead suggests that active, programmed cell death—such as apoptosis—may be induced as a biologically regulated response. These findings enable rational optimization of plasma treatment conditions for biological applications, thereby minimizing cellular damage during molecular delivery.

## Figures and Tables

**Table 1:** Parameters for each element used in the electrical equivalent circuit network (EECN) model.

Component	Parameter	Symbol	Value	Ref.
Cell	Height	*H*_c_	10 μm	[70]
	Permittivity	*ε*_c_	30*ε*_0_	[71]
	Conductivity	*σ*_c_	1 S/m	[71]
Membrane	Thickness	*T*_m_	7.5 nm	[72]
Buffer	Height	*H*_s_	0.1 mm	–
	Conductivity	*σ*_s_	0.172 S/m	–
Dish	Height	*H*_d_	1.5 mm	–
	Permittivity	*ε*_d_	2.4*ε*_0_	–

**Figure A1:**
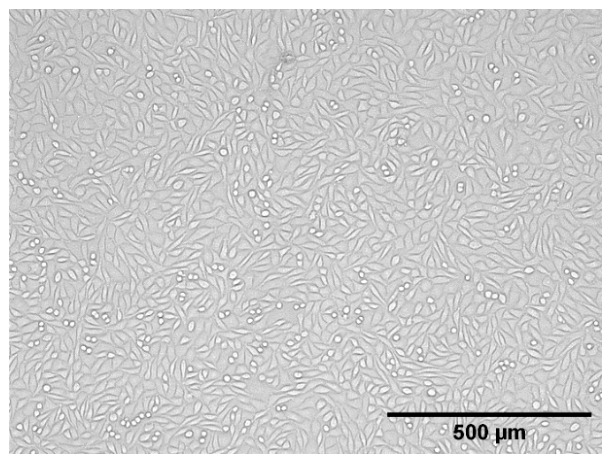
Representative pre-treatment bright-field image of cell confluency (≈100%).

## Data Availability

The raw data supporting the conclusions of this article will be made available by the authors on request.

## Abbreviations

The following abbreviations are used in this manuscript:

EECN	Equivalent electrical circuit network
EP	Electroporation
ER	Endoplasmic reticulum
FBS	Fetal bovine serum
GFP	Green fluorescent protein
IRE	Irreversible Electroporation
JSPS	Japan Society for the Promotion of Science
JST	Japan Science and Technology Agency
LNPs	Lipid Nanoparticles
mPTP	Mitochondrial permeability transition pore
mRNA	Messenger RNA
nsPEF	Nanosecond pulsed electric fields
PI	Propidium iodide
RNS	Reactive nitrogen species
RONS	Reactive oxygen and nitrogen species
ROS	Reactive oxygen species
SD	Standard deviation
